# The FITP: A Controlled Intermittent Running Test for Futsal Athletes

**DOI:** 10.3390/jfmk11030296

**Published:** 2026-07-27

**Authors:** João Nuno Ribeiro, Farzad Yousefian, Raul Filipe Bartolomeu, Faber Martins, Konstantinos Spyrou, Bruno Travassos, Carolina Vila-Chã, Pedro Tiago Esteves

**Affiliations:** 1 SPRINT, Sport Physical Activity and Health Research & Innovation Center, Polytechnic University of Guarda, 6300-559 Guarda, Portugal; bartolomeu@ipg.pt (R.F.B.); fabermartins@ipg.pt (F.M.); cvila-cha@ipca.pt (C.V.-C.); ptesteves@ipg.pt (P.T.E.); 2 FPF Academy, Federação Portuguesa de Futebol, 1495-218 Oeiras, Portugal; farzad.yousefian@ubi.pt (F.Y.); bruno.travassos@fpf.pt (B.T.); 3 CIDESD, Research Center in Sports Sciences, Health Sciences and Human Development, Department of Sport Sciences, Universidade da Beira Interior, 6200-001 Covilhã, Portugal; 4 Polytechnic University of Bragança, 5300-253 Bragança, Portugal; 5 Research Centre for Active Living and Wellbeing (LIVEWELL), Instituto Politécnico de Bragança, 5300-253 Bragança, Portugal; 6 UCAM Research Center for High Performance Sport, UCAM Universidad Católica de Murcia, 30107 Murcia, Spain; kspyrou@ucam.edu; 7 Faculty of Sport Sciences, Catholic University of Murcia (UCAM), 30107 Murcia, Spain; 8 Escola Superior de Desporto, Bem-Estar e Sistemas Biomédicos, Instituto Politécnico do Cávado e do Ave, 4750-810 Vila Frescainha, Portugal

**Keywords:** team sports, intermittent exercise, laboratory assessment, internal load, treadmill protocol, physiological monitoring

## Abstract

**Background**: Futsal is a high-intensity, intermittent team sport, yet laboratory protocols that reproduce its substitution-based running demands remain scarce. This study examined the physiological, neuromuscular, and perceptual responses to the Futsal Intermittent Treadmill Protocol (FITP), a laboratory-based protocol designed to replicate the intermittent high-intensity running demands of futsal. **Methods**: Ten male, national-level (Tier 3) futsal players completed four bouts of intermittent running (6–20 km·h^−1^) reflecting typical substitution patterns. Measurements included oxygen consumption (*VO*_2_), heart rate (HR), blood lactate concentration ([BLa^−^]), rating of perceived exertion (RPE), total quality recovery (TQR), and countermovement jump (CMJ) performance. Responses were analyzed using one-way repeated-measures ANOVA. **Results**: HR and %HRmax increased significantly across bouts (*p* < 0.001; η^2^*p* = 0.84 for both), reaching values typical of competitive matches. Relative *VO*_2_ and %*VO*_2_max remained stable, while RPE increased and TQR decreased progressively (*p* < 0.001; η^2^*p* = 0.59–0.62). Blood lactate levels and the CMJ mechanical variables showed no significant changes, indicating no systematic neuromuscular fatigue pattern. **Conclusions**: The FITP induces substantial cardiovascular and perceptual strain that is physiologically comparable to high-intensity futsal, while metabolic and neuromuscular responses remain stable. The protocol may provide a controlled, repeatable method for assessing intermittent running tolerance; however, optimizing its role in physical evaluation, top-up conditioning, and return-to-play monitoring will benefit from future research confirming its ongoing consistency and field application.

## 1. Introduction

Futsal is a high-intensity, intermittent team sport characterized by short recovery intervals and frequent in-match substitutions that result in highly demanding and fluctuating workloads. The constrained playing area (40 m × 20 m), the number of players involved (4v4 + GK), the structured timing of play with stoppages (2 × 20 min), fast-paced transitions, and strategic player substitutions generate a unique physical activity profile that differs markedly from other team sports [1,2,3].

Previous research has consistently reported that adult male futsal players exhibit mean heart rates between 85 and 90% of HRmax and blood lactate concentrations ranging from 6 to 12 mmol·L^−1^ during match performance [3,4,5,6]. Notably, Dos-Santos et al. (2020) [5] reported similar lactate values (8.4 vs. 8.2 mmol·L^−1^) in players completing, on average, two substitutions per half, suggesting that typical rotation patterns (i.e., player substitutions) do not markedly alter lactate responses during competition. Furthermore, top-level futsal players exhibit high values of oxygen consumption (*VO*_2_max), with mean values of approximately 62.8 mL·kg^−1^·min^−1^, while semiprofessional players demonstrate lower values, averaging 55.2 mL·kg^−1^·min^−1^ [7,8]. The rating of perceived exertion (RPE) has been shown to correlate with both *VO*_2_max and the overall training load accumulated over time [9]. Perceptual tools such as RPE and total quality recovery (TQR) are widely used to monitor internal load and recovery in team sports but have been only sparsely characterized during controlled, laboratory-based intermittent futsal tasks [10,11]; likewise, the integration of gas exchange, heart rate, blood lactate, and neuromuscular (CMJ) measures within a single standardized futsal protocol remains limited [10,12].

Previous studies have reported that elite futsal players usually cover between 3 and 5 km per match, with an average relative distance of 121 m·min^−1^ (range: 105–137 m·min^−1^) and spend approximately 5% and 12% of total playing time sprinting and performing high-intensity running, respectively [10,13].

These dynamic demands create a complex physiological and mechanical environment that challenges traditional testing approaches, especially when aiming to replicate match-specific loads in a controlled setting. For example, field-based tests, such as shuttle runs, or continuous incremental protocols, such as the Yo-Yo Intermittent Recovery Test or the Futsal Intermittent Endurance Test (FIET), have been shown to reflect sport-specific endurance capacity [10], yet often fail to reflect the match-rotation structure of futsal. From this perspective, such protocols show limitations in reproducing and interpreting the physical demands of futsal in controlled laboratory settings, which is a critical issue for accurate performance assessment, targeted training design, and even return-to-play decision-making. Critically, these tests index maximal aerobic or repeated-shuttle capacity but do not reproduce the discrete substitution–recovery (≈1:1) architecture of futsal, and their field-based nature precludes the simultaneous, standardized laboratory measurement of *VO*_2_, HR, [BLa^−^], and neuromuscular output.

Regarding the characterization of match demands according to player substitutions, previous studies suggest that elite athletes’ ability to sustain high-intensity efforts is closely related to the ratio between playing time and recovery periods. In futsal, these recovery periods result mainly from player rotations rather than natural stoppages in match play [14]. Ratios close to 1:1 are considered optimal for maintaining performance levels throughout the match [13,14]. In practice, coaches typically manage playing time in cycles of approximately 3 min on and 3 min off (effective time), corresponding to 4 to 6 action-recovery bouts of 6 min of total time per match [15]. This dynamic interchange format has important implications for fatigue management, particularly given the repeated bouts of maximal or near-maximal effort.

Despite the relevance of these dynamics, little is known about the physiological and neuromuscular responses that result from the simulation of successive substitutions under standardized conditions. Treadmill-based protocols stand as a promising solution, providing a controlled and standardized environment for continuous monitoring of physiological variables such as oxygen uptake (*VO*_2_), heart rate (HR), and blood lactate concentration while ensuring consistency across testing sessions [16]. This approach allows researchers to assess the impact of recovery during simulated substitutions, as well as the physiological responses of futsal players to high-intensity efforts in a match-like context.

As a result, there is a growing need for testing solutions that balance ecological validity with experimental control and that can capture both the physiological and perceptual responses relevant to high-intensity intermittent activity in futsal. Although intermittent treadmill simulations have been developed for other team sports [17,18], they are typically organized around fixed activity cycles rather than the substitution-driven structure that governs internal load in futsal, underscoring the need for a sport-specific solution.

The aim of the study was to analyze the physiological (*VO*_2_, HR, lactate), neuromuscular (countermovement jump; CMJ), and perceptual (RPE, TQR) responses elicited by a Futsal Intermittent Treadmill Protocol (FITP) and to explore its potential application as a laboratory-based tool to reproduce the intermittent high-intensity running demands characteristic of futsal match play. Accordingly, the present study constitutes a characterization of the FITP’s internal-load profile rather than a criterion validation against match play; the latter would require direct comparison with in-match data and was beyond the present scope. Based on previous literature and the structure of the protocol, we hypothesized that: (1) the physiological, neuromuscular, and perceptual responses elicited by the FITP would align with the intermittent high-intensity running demands typically observed in futsal; (2) the protocol would induce substantial physiological stress, as reflected by elevated *VO*_2_, HR, and [BLa^−^] compared to those reported during high-intensity periods of futsal competition; and (3) successive high-intensity bouts would result in increased RPE and, on an exploratory basis, changes in CMJ performance. Because treadmill running does not include the accelerations, decelerations, and changes in direction that constitute the principal source of eccentric neuromuscular load in futsal, we examined whether the cumulative cardiovascular and perceptual load of repeated high-intensity linear running would, by itself, be sufficient to elicit detectable neuromuscular changes. Metabolically, we expected [BLa^−^] to remain within the range reported for futsal match play rather than to accumulate progressively across bouts.

## 2. Materials and Methods

### 2.1. Experimental Approach to the Problem

The present experimental study aimed to develop and characterize a treadmill-based protocol—the Futsal Intermittent Treadmill Protocol (FITP)—designed to reproduce intermittent high-intensity running demands relevant to futsal within a controlled laboratory setting. The protocol was designed based on published time-motion analyses of elite-level futsal [2,3,19] and replicates typical player interchange rotation [14,15].

### 2.2. Subjects

A sample of 10 male futsal players (age: 24.0 ± 5.3 years; body mass: 70.6 ± 8.0 kg; height: 178.0 ± 7.6 cm; BMI: 22.3 ± 2.2 kg/m^2^; body fat: 11.1 ± 3.7%) participated in this study. The sample included four defenders, four wingers, and two pivots; nine players were right-leg dominant, and one was left-leg dominant, with an average of 14 ± 4.9 years of futsal experience. Participants trained four sessions per week (6 h·week^−1^) of futsal-specific training plus two resistance training sessions. Inclusion criteria were: (i) being a male futsal player competing at the national level (Tier 3 according to the participant classification framework [20]); (ii) being actively training and competing at the time of the study; and (iii) being free of injury and illness. Players were excluded if they had sustained any musculoskeletal injury or reported any health limitation in the three months preceding data collection or if they presented any contraindication to high-intensity exercise. Every athlete provided written informed consent, and the study was conducted in accordance with the Declaration of Helsinki and approved by the Ethics Committee of the Polytechnic Institute of Guarda (No. 9/2024; approval date 20 June 2024).

### 2.3. Procedures

**Figure 1:**
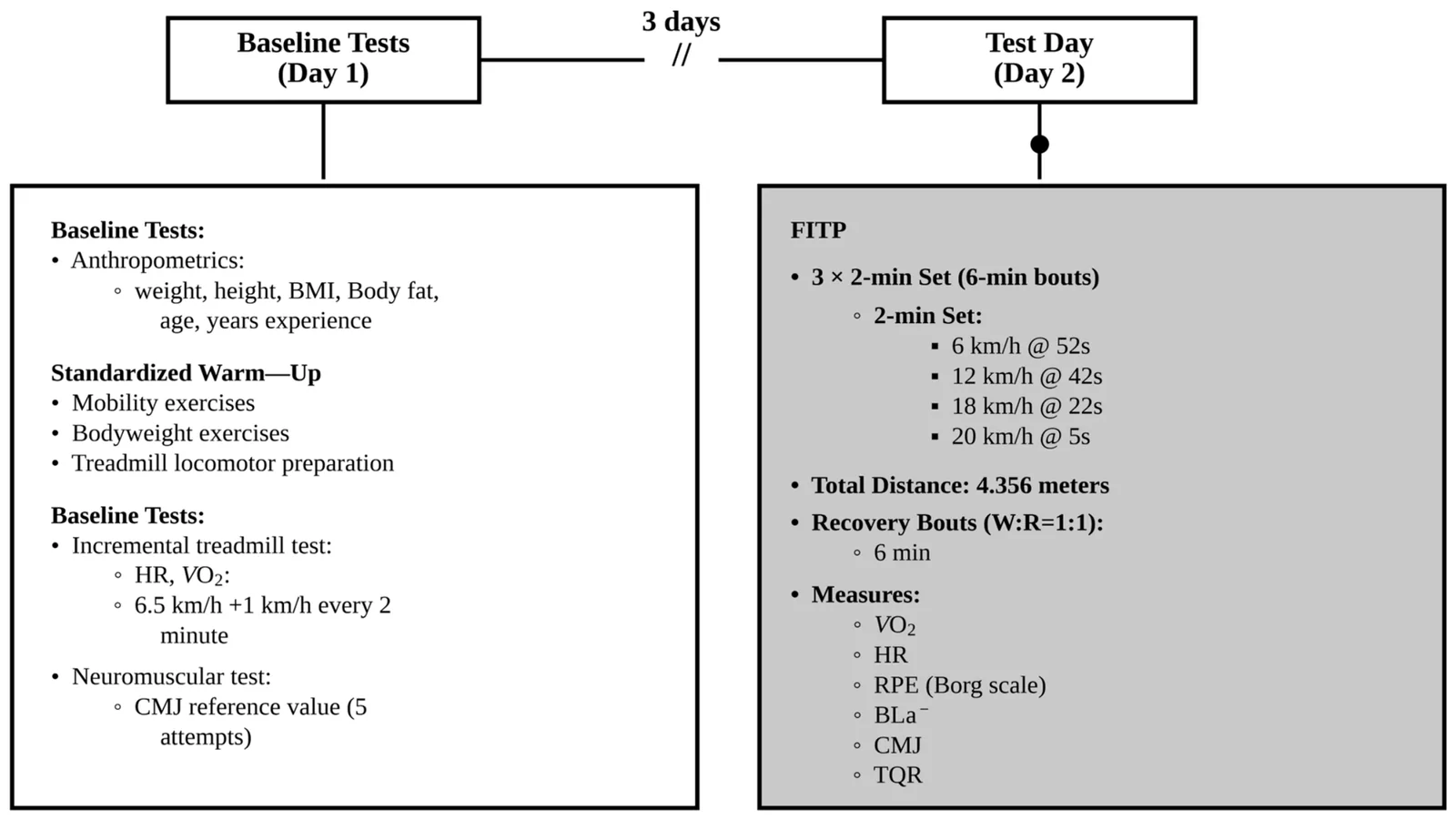
Schematic representation of the testing protocol. BMI, body mass index, body fat, CMJ, countermovement jump; TQR, total quality recovery; RPE, rating of perceived exertion; BLa^−^, blood lactate; HR, heart rate; *VO*_2_, oxygen consumption; W:R, work-to-rest ratio.

The testing protocol consisted of one baseline assessment followed by a single experimental testing session (the FITP), separated by a 3-day interval (Figure 1). All testing sessions were conducted in a laboratory and scheduled at the same time of day (±1 h) for each participant to minimize circadian rhythm variations. Participants attended the laboratory in a 2 h post-absorptive state, following a self-reported 48 h period of abstinence from vigorous exercise and alcohol, and were instructed to refrain from consuming caffeine for 24 h prior to all experimental trials.

Day 1 comprised baseline assessments and familiarization with the experimental protocol. Anthropometric measurements included standing height, recorded using a portable stadiometer (Seca 213, Seca GmbH, Hamburg, Germany; nearest 0.1 cm), and body composition (body mass index and body fat percentage), assessed using a bioelectrical impedance analyzer (InBody 270, InBody Co., Seoul, Republic of Korea). Both instruments have published evidence of good reliability/validity for field use [21,22].

Following this, athletes were instructed by a certified strength and conditioning coach to perform a standardized warm-up protocol, consisting of mobility exercises and progressive bodyweight exercises (air squats, lunges, hip bridges, calf raises, and jump squats). The warm-up also included a series of locomotor activities designed to replicate the distribution and frequency of speed changes inherent to the experimental protocol, thereby promoting neuromuscular activation and movement-specific readiness.

Immediately after, athletes performed five CMJ to establish an individual reference mean. All jumps were performed according to the literature, with athletes keeping their hands on their hips and being verbally encouraged to jump as high as possible [23].

Participants then completed an incremental submaximal treadmill test (H/P/Cosmos Pulsar 4.0; H/P/Cosmos Sports and Medical GmbH, Nussdorf-Traunstein, Germany) to estimate maximal aerobic capacity [24]. The test started at 6.5 km·h^−1^, with speed increasing by 1 km·h^−1^ every 2 min until the participant reached 80–85% of their theoretical HRmax. The treadmill incline was set at 1%, a standard procedure shown to approximate the energetic cost of running and avoid the mechanical underestimation of efforts [25]. The FITP was administered on Day 2.

### 2.4. Futsal Intermittent Treadmill Protocol (FITP)

**Figure 2:**
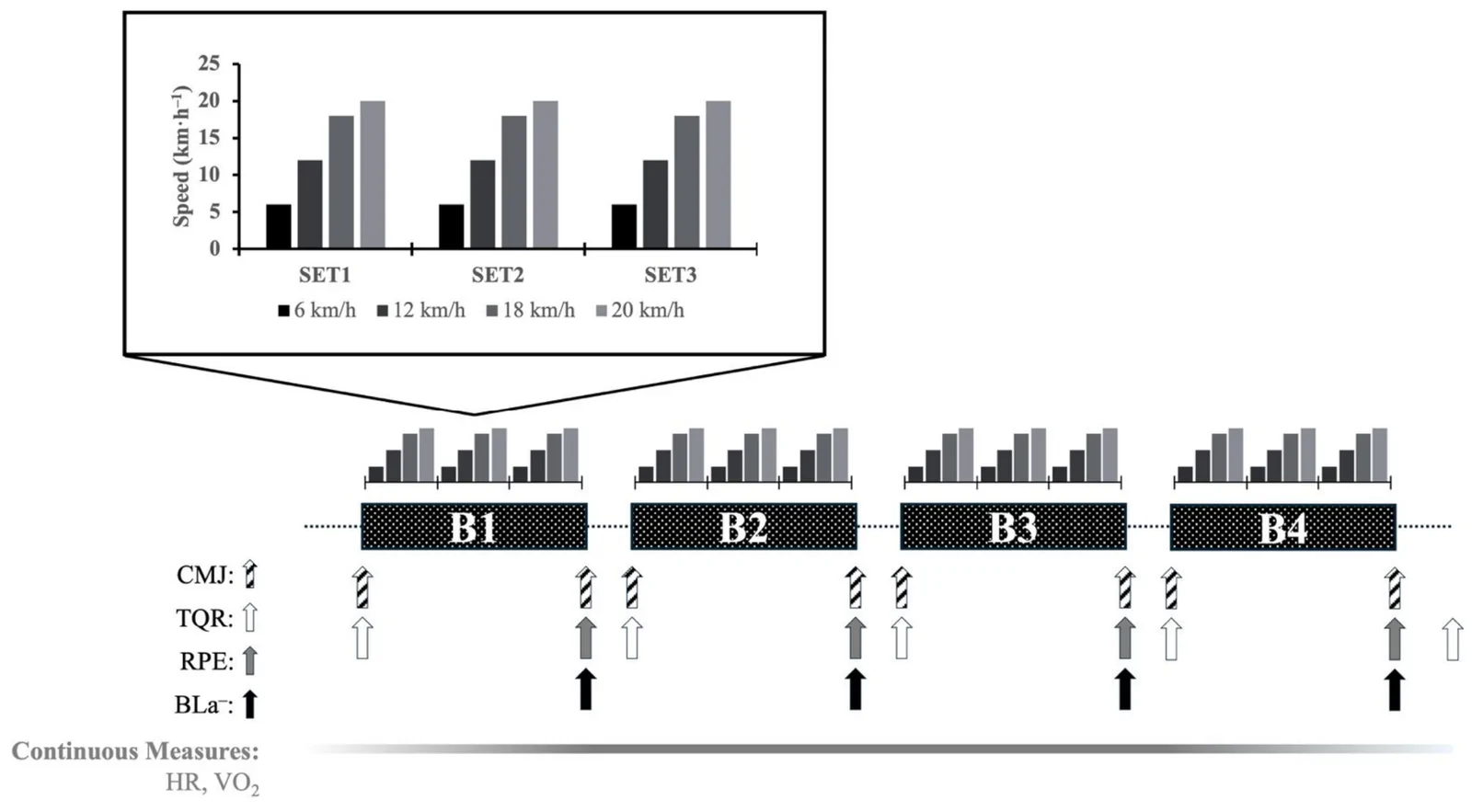
The Futsal Intermittent Treadmill Protocol (FITP), consisting of four running bouts (B1–B4). Each bout comprised three sets (SET1–SET3). Within each set, running speed increased progressively from 6 to 20 km·h^−1^, with each speed maintained for 52 s (6 km·h^−1^), 42 s (12 km·h^−1^), 22 s (18 km·h^−1^), and 5 s (20 km·h^−1^). The progression restarted at 6 km·h^−1^ at the beginning of each new set. Physiological (*VO*_2_, HR, BLa^−^), neuromuscular (countermovement jump; CMJ), and perceptual (RPE, TQR) measures collected during the FITP are also indicated.

The FITP (Figure 2) consisted of four running bouts. Each bout comprised three sets (SET1–SET3). Within each set, running speed increased progressively from 6 to 20 km·h^−1^ (6, 12, 18, and 20 km·h^−1^), with the speed progression restarting at 6 km·h^−1^ at the beginning of each new set. Specific durations and speed transitions are illustrated in Figure 2. These speed zones are consistent with futsal time-motion analyses and reflect the intermittent nature of futsal, characterized by frequent transitions between low- and moderate-intensity running and brief high-speed and sprinting actions [2,3]. The time spent at each speed was set so that the distance accumulated within each speed zone quantitatively reproduced the mean per-zone distances reported for elite futsal match play [2], thereby grounding the protocol in match-derived speed zone distributions. During the protocol, one researcher was responsible for programming the treadmill speed changes, controlling the timing, and providing verbal instructions and encouragement to the athlete. At each speed transition, the athlete was required to step onto the treadmill side rails with both feet and re-enter immediately once the target speed was set. This maneuver was standardized across participants and sessions by the same researcher, who supervised every transition and provided consistent verbal cues, and participants were familiarized with it on Day 1. The brief straddling of the belt was required to permit the controlled speed changes and the continuous laboratory measurement of pulmonary gas exchange. The protocol was structured into four bouts (B1–B4), reflecting the typical number of substitutions performed in competitive futsal [15]. Before each bout, players performed two CMJs and reported their TQR. At the end of each bout, they again completed two CMJs, reported RPE, and [BLa^−^] was measured, followed by a six-minute passive recovery period (seated rest), resulting in a work-to-rest ratio of 1:1 [15]. Within each set, the four speeds were maintained for 52 s (6 km·h^−1^), 42 s (12 km·h^−1^), 22 s (18 km·h^−1^), and 5 s (20 km·h^−1^), giving an effective running time of 121 s per set and ≈6 min of running per bout across the three sets; the belt was not stopped during the brief side-rail transitions.

Across the entire protocol, participants covered a total distance of 4356 m, closely matching the average total distance reported during match play [3]. Figure 2 illustrates both the performance and recovery indicators assessed and the specific time points at which data were collected throughout the protocol (HR, *VO*_2_, BLa^−^, CMJ, RPE, and TQR).

### 2.5. Physical Measurements

#### 2.5.1. Cardiorespiratory Measures

Cardiorespiratory variables were continuously recorded using a breath-by-breath spiroergometric system (MetaMax 3B, Cortex Biophysik, Leipzig, Germany) synchronized with a chest-strap heart rate monitor (H10, Polar Electro, Kempele, Finland), demonstrating both high reliability and stability [26,27]. Data were transmitted telemetrically and monitored in real time using the manufacturer’s software. The spiroergometric system was calibrated before each test against ambient air and a known-volume 3 L syringe according to the manufacturer’s specifications, and all sessions were performed in a temperature-controlled laboratory.

At each laboratory visit, resting *VO*_2_ and HR were obtained during the final minute of a 10 min supine rest period. On Day 1, participants completed an intermittent, progressive running protocol to characterize the *VO*_2_–HR relationship up to ~80–85% of their age-predicted maximal HR (HRmax = 208 − 0.7 × age) [28]. All HR values were expressed as a percentage of heart rate reserve (%HRres), calculated as shown in Equation (1):

%HRres = [(HR − HRrest)/(HRmax − HRrest)] × 100(1)

Based on established evidence supporting the relationship between %HRres and %*VO*_2_ reserve (%*VO*_2_res) [29], *VO*_2_max was extrapolated via linear regression between %*VO*_2_res and absolute *VO*_2_, adding *VO*_2_rest to the predicted maximal value [30,31,32,33]. During Days 2 and 3, %HRres and %*VO*_2_res were computed continuously using the same procedures. The estimated *VO*_2_max averaged 58.6 ± 5.1 mL·kg^−1^·min^−1^ (range 49–67). The 80–85% HRmax termination criterion was adopted to reduce participant burden at the end of the competitive season. Growing evidence demonstrates that submaximal protocols offer highly reliable estimates of aerobic capacity without the neuromuscular fatigue of maximal testing. While direct maximal testing remains the gold standard, this submaximal estimation provided a valid, practical, and safe approximation of the athletes’ *VO*_2_max [34].

#### 2.5.2. Metabolic Measures

Blood lactate (BLa^−^) was measured as an indicator of the physiological stress imposed by the high-intensity intermittent exercise protocol, reflecting the contribution of anaerobic glycolysis and the accumulation of fatigue during the protocol. Capillary blood samples (~5 µL) were collected from the participant’s middle finger and immediately analyzed using a portable lactate analyzer (Lactate Pro2, Arkray, Kyoto, Japan), following the manufacturer’s instructions. This device has been previously validated and shown to be reliable for these measures [35].

#### 2.5.3. Perceived Measures

Perceptual measures, RPE [36] and TQR [37], were collected in parallel with the BLa^−^ measures. RPE was recorded upon completion of each bout (B1–B4), and TQR was collected after each recovery period. Participants were shown an A4-sized sheet displaying the CR10 RPE scale and asked to indicate their perceived effort, where 0 corresponded to ‘no exertion’ and 10 to ‘maximal exertion’. After each 6 min recovery period, participants were presented with an A4-sized sheet displaying the TQR scale and asked to select a value from 6 to 20, where 6 indicated feeling not recovered at all and 20 indicated very, very good recovery.

#### 2.5.4. Neuromuscular Measures

CMJ performance was assessed using a 60 cm × 50 cm force platform (KiJump 9229A, Kistler, Winterthur, Switzerland), the criterion instrument for kinetic assessment [38]. Participants performed repeated CMJ efforts, and a trial was considered valid only when the landing was stable and within the platform boundaries. Invalid attempts were repeated until two valid and independent jumps were obtained. The two valid jumps were separated by approximately 10 s of rest, and the mean of the two jumps was used for analysis; jump height was derived from the vertical take-off velocity (impulse–momentum method), with ground reaction forces sampled at 1000 Hz. Jump height (cm), concentric duration (ms), concentric peak power (W), eccentric duration (ms), and eccentric peak power (W) were extracted using the manufacturer’s proprietary software (MARS 5.0, Kistler, Switzerland). Furthermore, the modified reactive strength index (RSI-M) was calculated as jump height divided by the time to take off, with the latter defined as the total contraction time from the onset of the CMJ to take-off (i.e., the combined eccentric and concentric phase durations). Variable selection was based on previous research identifying these metrics as reliable indicators of neuromuscular performance and sensitive markers of acute fatigue [39,40,41].

### 2.6. Statistical Analyses

Data are presented as mean ± SD and coefficient of variation (CV) with 95% confidence intervals. Normality was assessed using the Shapiro–Wilk test and sphericity with Mauchly’s test; Greenhouse–Geisser corrections were applied when necessary. Differences between bouts and protocol phases (pre vs. post) were examined using repeated-measures ANOVA, followed by Bonferroni-adjusted post hoc comparisons. Specific comparisons between baseline and each subsequent phase were performed using paired *t*-tests (or Wilcoxon tests when assumptions were violated). Effect sizes were reported as partial η^2^ (ANOVA) and Cohen’s *d* (paired comparisons). The significance level was set at *p* < 0.05. Analyses were conducted in Jamovi (version 2.3.28.0). All assumptions were checked and met unless otherwise specified. Where sphericity was violated (Mauchly’s test), Greenhouse–Geisser-corrected values are reported. Missing data were handled by analyzing the available complete cases for each repeated-measures model. A sensitivity power analysis (G*Power 3.1; repeated-measures ANOVA, within factors; α = 0.05, power = 0.80, four measurements, ρ = 0.5) indicated that the sample (*N* = 10) was adequate to detect large effects (f ≥ 0.40; partial η^2^ ≥ 0.14).

## 3. Results

### 3.1. Physiological Responses

**Figure 3:**
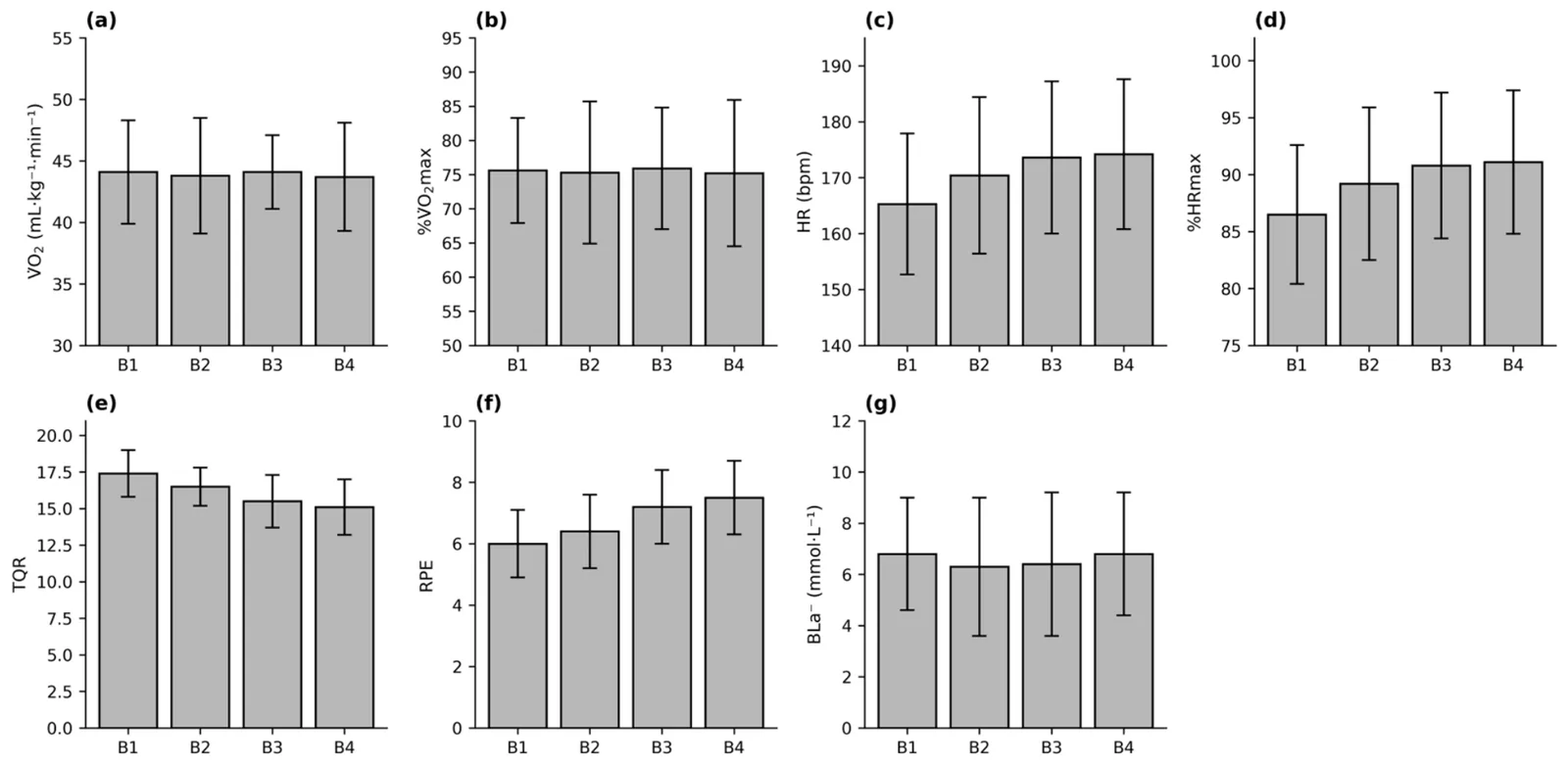
Bout-to-bout progression of physiological and perceptual variables across the four exercise bouts (B1–B4) of the FITP. (**a**) *VO*_2_, (**b**) %*VO*_2_max; (**c**) HR; (**d**) %HRmax; (**e**) TQR; (**f**) RPE; (**g**) BLa^−^.

Mean values and coefficients of variation (%CV) for all physiological variables across bouts are presented in Table 1, and their bout-to-bout progression is illustrated in Figure 3.

Repeated-measures ANOVA revealed no significant changes in relative *VO*_2_ or %*VO*_2_max across bouts (*p* > 0.05, η^2^*p* = 0.18, 95% CI [0.00, 0.06]), with moderate intra-individual variability (<15%). Heart rate increased significantly across bouts, with Bout 1 (B1) lower than B2–B4 and B2 lower than B3–B4 (*p* < 0.001, η^2^*p* = 0.84, 95% CI [0.66, 0.89]); no difference was observed between B3 and B4. Similarly, %HRmax showed a significant main effect, with progressive increases up to B3 and stabilizing in B4 (*p* < 0.001, η^2^*p* = 0.84, 95% CI [0.66, 0.89]).

### 3.2. Perceptual Responses

Perceived recovery (TQR) decreased significantly across bouts (*p* < 0.001, η^2^*p* = 0.59, 95% CI [0.27, 0.70]), with lower values in B4 compared to B1 and B2 (Table 1). Conversely, perceived exertion (RPE) increased progressively, with significantly lower values in B1 compared to B3 and B4 and in B2 compared to B4 (*p* < 0.001, η^2^*p* = 0.62, 95% CI [0.32, 0.73]). These patterns are illustrated in Figure 3.

### 3.3. Metabolic Response

Blood lactate concentrations (BLa^−^) remained stable across bouts (*p* > 0.05, η^2^*p* = 0.01, 95% CI [0.00, 0.06]), despite substantial intra-individual variability (CV = 32–44%).

### 3.4. Neuromuscular Responses

Neuromuscular variables across baseline, pre-, and post-bout measurements are presented in Table 2. CMJ height showed no significant main effect of bout (F = 1.32, *p* = 0.291, η^2^*p* = 0.14) or of phase (pre vs. post; t = −0.41, *p* = 0.690, d = −0.14). Similarly, no significant bout or phase effects were observed for concentric duration (*p* = 0.210, d = 0.45), concentric peak power (*p* = 0.132, d = −0.56), eccentric duration, or eccentric peak power (all *p* > 0.05). Effect sizes were small to moderate throughout (d = −0.56 to 0.45), with values fluctuating around baseline without a systematic bout- or fatigue-related pattern.

## 4. Discussion

The present study aimed to analyze the physiological, neuromuscular, and perceptual responses elicited by the FITP and to explore its potential use as a laboratory-based tool to reproduce intermittent high-intensity running demands characteristic of futsal and support athlete monitoring. Overall, the results partially confirm our initial hypotheses. The FITP induced substantial cardiovascular and perceptual strain consistent with competitive futsal, while metabolic (blood lactate) and neuromuscular (CMJ) responses showed more stable patterns, suggesting that the protocol reproduces key aspects of the internal physiological load of high-intensity futsal.

Heart rate responses during the FITP were high and increased progressively from B1 to B3, stabilizing thereafter, with %HRmax values approaching those typically observed in competitive futsal match play (≈85–90% HRmax) [7,13,42]. This rise-and-plateau pattern aligns with the cardiovascular drift and cumulative autonomic strain characteristic of intermittent high-intensity exercise [16,43]. The absence of changes in relative *VO*_2_ and %*VO*_2_max across bouts, alongside moderate intra-individual variability, indicates that cardiorespiratory stress increased rapidly early in the protocol and was subsequently maintained at a steady-state level despite the continued rise in HR. Similar HR–*VO*_2_ dissociations have been reported in team-sport intermittent tests and match play, reflecting thermoregulatory and autonomic influences on HR beyond metabolic load [44,45]. From a validity standpoint, the combination of elevated HR, stable elevated *VO*_2_, and repeated speed changes suggests that the FITP reproduces the internal high-intensity intermittent aerobic–anaerobic load of futsal [7,13]. The protocol appears to impose sustained cardiorespiratory stress comparable to in-match demands, supporting its suitability as a controlled and repeatable tool for assessing physiological responses and its potential for monitoring longitudinal adaptation once its reliability and criterion validity have been established.

Contrary to our second hypothesis, [BLa^−^] did not increase systematically across bouts, despite clear rises in HR and RPE. Previous time-motion and physiological studies in futsal typically report mean lactate values of ~4–8 mmol·L^−1^, with substantial inter-individual and intra-match variability [1,5,13]. The relatively high coefficient of variation observed in the current study (32–44%) aligns with this known variability and likely reflects inter-individual differences in glycolytic contribution, muscle fiber recruitment, and lactate clearance among players [46,47]. The limited variation in [BLa^−^] across bouts may be partially influenced by the displacement characteristics of the FITP, which rely predominantly on linear running. Although futsal involves more complex multidirectional actions [2,3], the FITP still provides a controlled stimulus that captures key metabolic aspects of high-intensity intermittent play. Because the observed values (6.3–6.8 mmol·L^−1^) fall within the range reported during futsal match play [5], their stability across bouts is consistent with a predominantly aerobic energy contribution punctuated by brief anaerobic efforts. This response produced a quasi-steady state in [BLa^−^], likely facilitated by the 6 min inter-bout recovery period that permitted partial lactate clearance, alongside the largely linear running profile, which limited glycolytic recruitment. This pattern is also consistent with the unlimited substitutions permitted in futsal, which create a work-to-rest ratio close to 1:1 [15], allowing players to intermittently recover and thereby attenuating progressive lactate accumulation during match play. Similar patterns have been reported in intermittent field tests where intense work is interspersed with short recovery periods [9,48]. Overall, these findings indicate that although lactate responses during the FITP fall within the broad [BLa^−^] range previously observed in futsal, the marked inter-individual variability reinforces the need to interpret lactate values cautiously and in combination with other internal load markers.

The progressive increase in RPE and the concomitant reduction in TQR across bouts support our third hypothesis and align with previous findings in futsal and other court-based team sports [9,48]. RPE displayed a clear dose–response to accumulating work, with the highest values in the final bout, consistent with evidence that RPE integrates metabolic, cardiovascular, and neuromuscular inputs into a single perceptual index of strain [36,49]. Likewise, the decline in perceived recovery reflects a growing mismatch between task demands and perceived capacity to sustain performance, a pattern also observed during congested match schedules and repeated high-intensity sessions [9]. From an applied perspective, these perceptual responses reinforce the value of simple tools such as RPE and TQR in futsal-specific testing. The increase in RPE despite stable *VO*_2_ and the absence of a systematic lactate response suggest that perceptual markers may be more sensitive to the cumulative psycho-physiological burden of intermittent exercise than isolated physiological variables [50,51]. Integrating RPE and TQR with HR-derived metrics during the FITP may therefore enhance internal load assessment and support more individualized training prescriptions.

Contrary to our expectation of progressive neuromuscular fatigue, CMJ height and most jump-derived mechanical variables (RSI-M, concentric and eccentric peak power, and eccentric duration) remained unchanged across bouts and between pre- and post-measures. The small-to-moderate, bidirectional effect sizes relative to baseline, with fluctuations between potentiation and fatigue, are consistent with the complex interaction of central and peripheral mechanisms during intermittent high-intensity exercise [52,53].

In the present study, two factors may explain the absence of systematic CMJ decrements. First, the overall volume and density of the FITP appear sufficient to elicit substantial cardiovascular and perceptual strain but insufficient to generate marked neuromuscular fatigue. Second, the treadmill-based nature of the protocol, which lacks mechanically demanding efforts such as accelerations, decelerations, and changes in direction, likely reduced the eccentric loading typically associated with neuromuscular impairments in intermittent court-based sports [54]. Overall, these results demonstrate that the FITP elicits an internal physiological and perceptual load consistent with the intermittent high-intensity profile of futsal while producing minimal neuromuscular disruption, thereby providing a clear characterization of athletes’ responses to repeated high-intensity running in a controlled laboratory setting.

Some limitations should be acknowledged. First, despite the high level of the participants, the small sample size should be acknowledged. Second, because no maximal tests were performed, HRmax and *VO*_2_max had to be estimated from submaximal procedures, which reduces participant burden but introduces potential error when interpreting maximal cardiovascular responses [31,55,56]. Third, the FITP does not fully reproduce the mechanical complexity of futsal match play, particularly the eccentric loading associated with frequent changes in direction, which may influence neuromuscular responses and impact on ecological validity. Furthermore, no concurrent external-load data were collected for direct match comparison. Fourth, the sample included only male players, limiting generalizability to female athletes. Finally, the test–retest reliability and criterion validity of the FITP were not established in this study. Furthermore, FITP does not capture the technical, tactical, and decision-making demands of futsal, which co-determine competitive performance, it does not inform on the physiological or neuromuscular adaptations that may result from a match.

These factors should be considered when interpreting the findings. Future research with larger cohorts should validate this protocol against direct match data, and it would be valuable to design and test a protocol based on peak match demands.

## 5. Practical Applications

The FITP offers several practical applications for performance staff: (a) it provides a controlled and standardized laboratory protocol that can be applied at any point in the season to obtain consistent physiological and perceptual indicators that are difficult to isolate in field environments, which, once its reliability and criterion validity are established, may support physical assessment beyond pre-season testing; and (b) with further validation in injured athletes, its reproducible intermittent stimulus may in the future contribute to return-to-play frameworks by allowing practitioners to compare an athlete’s %HRmax, *VO*_2_, RPE, and TQR responses with pre-injury or team reference values to judge readiness for progression. As preliminary and illustrative benchmarks that would require prospective validation, indicators such as stabilization of HR across the final bouts (B3–B4), TQR ≥ 15, RPE ≤ 8, and, provided a reliable countermovement-jump measure is available, CMJ height within ~90% of the individual pre-injury baseline could be explored as readiness criteria; and (c) it can serve as a structured top-up conditioning tool for players with low match exposure, with the option of adding brief court-based actions when a more match-representative load is required.

## 6. Conclusions

The FITP induces substantial cardiovascular and perceptual strain consistent with high-intensity futsal, while metabolic and neuromuscular responses remain stable. The protocol provides a controlled and standardized method for assessing intermittent running tolerance. Consequently, it may serve as a promising tool for physical evaluation, top-up conditioning, and potential return-to-play processes in futsal athletes, although further validation, including the establishment of its test–retest reliability and criterion validity, and studies in injured cohorts, is warranted, with neuromuscular status assessed alongside the physiological and perceptual responses.

## Figures and Tables

**Table 1:** Mean values (±SD), coefficient of variation (%), and 95% confidence intervals of physiological and perceptual variables across the four exercise bouts.

Variable	Bout 1	Bout 2	Bout 3	Bout 4
*VO*_2_ (mL/kg/min)	44.1 ± 4.3 (10%) [40.9, 47.3]	43.8 ± 4.7 (11%) [40.2, 47.4]	44.1 ± 3.0 (7%) [41.8, 46.4]	43.7 ± 4.4 (10%) [40.3, 47.0]
%*VO*_2_max	75.6 ± 7.7 (10%) [69.7, 81.6]	75.3 ± 10,4 (14%) [67.3, 83.3]	75.9 ± 8.9 (12%) [69.1, 82.7]	75.2 ± 10.7 (14%) [67.0, 83.4]
HRavg	165.3 ± 12.6 (8%) ^¶^ [155.6, 175.1]	170 ± 14 (8%) ^β^ [159.7, 181.2]	173.6 ± 14 (8%) [163.1, 184]	174.2 ± 13.4 (8%) [163.9, 184.5]
%HRMax	86.5 ± 6.1 (7%) ^¶^ [81.8, 91.2]	89.2 ± 6.7 (8%) ^β^ [84.0, 94.5]	90.8 ± 6.4 (7%) [85.9, 95.7]	91.1 ± 6.3 (7%) [86.3, 96.0]
TQR	17.4 ± 1.6 (9%) * [16.3, 18.5]	16.5 ± 1.3 (8%) * [15.6, 17.4]	15.5 ± 1.8 (12%) [14.2, 16.8]	15.1 ± 1.9 (13%) [13.7, 16.5]
RPE	6.0 ± 1.1 (18%) ^†,^* [5.2, 6.8]	6.4 ± 1.2 (18%) * [5.6, 7.2]	7.2 ± 1.2 (16.8%) [6.3, 8.1]	7.5 ± 1.2 (16%) [6.7, 8.3]
BLa^−^	6.8 ± 2.2 (32%) [5.2, 8.3]	6.3 ± 2.7 (42%) [4.4, 8.2]	6.4 ± 2.8 (44%) [4.4, 8.4]	6.8 ± 2.4 (36%) [5.0, 8.5]

Note: ^¶^ significantly different from all other bouts (*p* < 0.001); ^β^ significantly different from Bout 3 and Bout 4 (*p* < 0.001); ^†^ significantly different from Bout 3 (*p* < 0.05); * significantly different from Bout 4 (*p* < 0.05).

**Table 2:** Mean values (±SD) of countermovement jump neuromuscular variables at baseline and at pre- and post-measurements across the four exercise bouts.

	Phase	Bout 1		Bout 2		Bout 3		Bout 4	
Variable	Baseline	Pre	Post	Pre	Post	Pre	Post	Pre	Post
CMJ-H (cm)	40.9 ± 8.7	39.3 ± 8.9	38.4 ± 7.8	40.8 ± 8.0	36.7 ± 5.5	39.3 ± 6.2	40.4 ± 13.3	39.3 ± 10.5	43.8 ± 5.4
RSI-M	0.53 ± 0.13	0.49 ± 0.15	0.48 ± 0.11	0.51 ± 0.09	0.47 ± 0.10	0.50 ± 0.09	0.52 ± 0.21	0.49 ± 0.12	0.62 ± 0.24
CDur (ms)	268 ± 42	278 ± 43	278 ± 34	285 ± 46	273 ± 35	284 ± 50	272 ± 39	286 ± 65	284 ± 56
CPP (w)	3781 ± 539	3597 ± 499	3731 ± 660	3768 ± 639	3664 ± 434	3723 ± 576	3882 ± 923	3751 ± 746	4147 ± 962
EDur (ms)	522 ± 79	546 ± 91	534 ± 59	519 ± 70	525 ± 80	511 ± 79	525 ± 95	510 ± 63	485 ± 63
EPP (w)	1238 ± 510	1162 ± 510	1160 ± 427	1185 ± 616	1275 ± 546	1237 ± 587	1239 ± 529	1239 ± 610	1188 ± 485

Note: CMJ-H = countermovement jump; RSI-M = modified reactive strength index; CDur = concentric duration; CPP = concentric peak power; EDur = eccentric duration; EPP = eccentric peak power.

## Data Availability

The data presented in this study are available from the corresponding author upon reasonable request.

## Abbreviations

The following abbreviations are used in this manuscript:

FITP	Futsal Intermittent Treadmill Protocol
HR	Heart rate
*VO*_2_	Oxygen consumption
RPE	Rating of perceived exertion
TQR	Total quality recovery
[BLa^−^]	Blood lactate concentration
CMJ	Countermovement jump
RSI-M	Modified reactive strength index
CV	Coefficient of variation
